# The birds of Genome10K

**DOI:** 10.1186/2047-217X-3-32

**Published:** 2014-12-11

**Authors:** Stephen J OBrien, David Haussler, Oliver Ryder

**Affiliations:** Theodosius Dobzhansky Center for Genome Bioinformatics, St. Petersburg State University, St. Petersburg, 199004 Russia; Oceanographic Center, Nova Southeastern University, Ft Lauderdale, FL 33004 USA; Department of Biomolecular Engineering, University of California, Santa Cruz, CA 95064 USA; San Diego Zoo Institute for Conservation Research, Escondido, CA 92027 USA

**Keywords:** Birds, Aves, Avian, Vertebrate, Genomics, Genome10K, Phylogenomics, Avian phylogenomics project

## Abstract

Everyone loves the birds of the world. From their haunting songs and majesty of flight to dazzling plumage and mating rituals, bird watchers – both amateurs and professionals - have marveled for centuries at their considerable adaptations. Now, we are offered a special treat with the publication of a series of papers in dedicated issues of *Science, Genome Biology* and *GigaScience* (which also included pre-publication data release). These present the successful beginnings of an international interdisciplinary venture, the Avian Phylogenomics Project that lets us view, through a genomics lens, modern bird species and the evolutionary events that produced them.

## Background

*"With the same unity of purpose shown for the Human Genome Project, we can now contemplate reading the genetic heritage of all species, beginning today with the vertebrates." G10KCOS-2009.*

**Figure 1:**
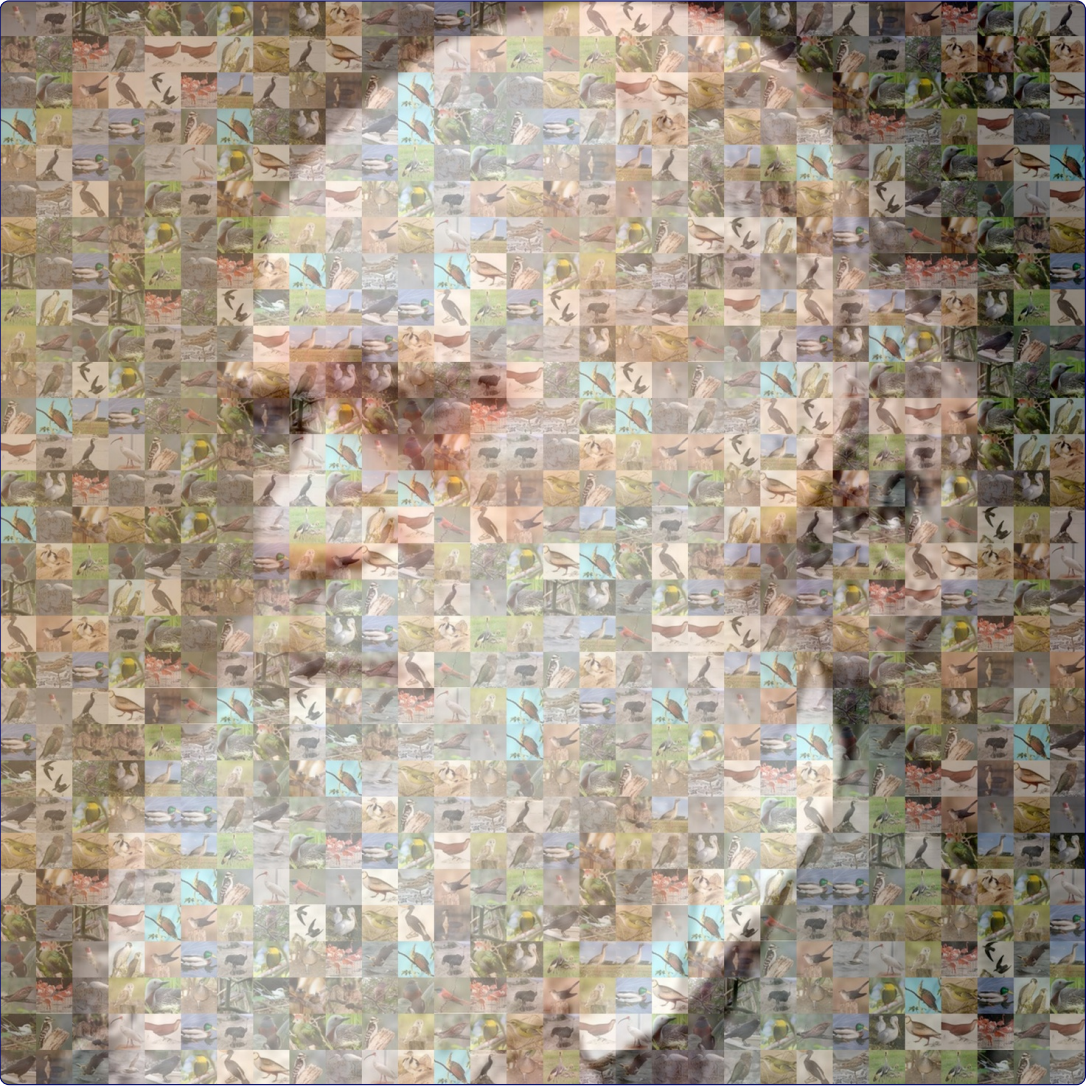
**A wealth of discoveries and genome resources are coming from the Avian Phylogenomics project documented in some 35 papers published simultaneously this week (or soon and now in press) in** ***Science*** **,** ***GigaScience*** **,** ***Genome Biology*** **, and in other familiar journals.** The reports feature many important advances including genomic inference around molecular phylogenetics, comparative genomics, penguin adaptation to a cold aquatic environment, pelican optics, losing their teeth, convergence of flight for birds and bats, crested ibis conservation, and a Crocodilian genome to recapitulate ancestral archosaur genomes. Figure courtesy of Rob Davidson.

The complete genome sequences of 48 avian species —crow, duck, falcon, parakeet, crane, egret, ibis, woodpecker, ostrich, sand grouse, eagles, finches, and many many more— all carefully selected for phylogenetic breadth and diversity from each of 30 Neoaves orders (comprising 95% of living bird species) have been assembled, posted and inspected for genetic determinates of more than a dozen avian capabilities and specialties [1, 2]. The detail and density of new insights is remarkable and unprecedented in comparative vertebrate genomics, even as the hopes of widespread comparative assessment are widely heralded. These publications and the ongoing efforts of the Avian Phylogenomics Project realize these anticipations in multiple dimensions (see Figure 1).

Modern birds trace their origins to the Jurassic epoch as reptilian ancestors of crocodile and feathered relatives emerged from the dinosaur lineages. When a 10 km-wide meteorite struck the Yucatan Peninsula 66 million years ago, the collision blackened the planet, terminating the vast and successful dinosaur world dominance [3]. The cataclysm is widely believed to have opened new ecological niches thereby allowing the emergence of terrestrial mammal diversification and their (and our) subsequent world domination. At the same time the avian ancestral species found the skies free from Archaeopteryx. The reduced ecological constraints precipitated a contemporaneous star-like (big-bang) evolutionary radiation into the most specious terrestrial vertebrate class, Aves, which today comprises some 10,500 living bird species [4]. This detailed history, enriched by morphological, behavioral, molecular and paleontological data has produced fascinating opportunities to examine evolutionary processes, neuroscience, and developmental biology, even as the reduction of species —largely through anthropogenic agency— has brought them into conservation focus. However, with all of this intriguing material for study, relatively few birds have enjoyed genomic attention so far: the chicken, turkey and zebra finch were the only sequenced bird genomes [5–7] when the Avian Group began. Today’s collection of reports changes that — for the better.

## Main text

The Avian Genome Project is an audacious outgrowth of the Genome 10K project (G10K), which was launched in 2009 ( http://genome10k.org). G10K is a consortium of genome scientists who aim to facilitate the whole genome sequence and analyses of 10,000 vertebrate species [8]. Within the G10K, the Avian Phylogenomics Group — which the bird collaborators call themselves— are led by Guojie Zhang (BGI), Erich Jarvis (Duke University), and Tom Gilbert (Natural History Museum of Denmark). They joined the Genome10K project to recruit over 200 collaborative experts in avian and archosaur genomics to create an international partnership addressing two principal issues: the phylogenetic hierarchy of the avian radiation and the bases for flight and function adaptations that comparative genomics would offer^a^. At this point in the G10K project, scientists across the globe have nearly completed whole-genome sequencing for over 259 vertebrate species (Table 1) — and 45 of these, now completed, are birds: a first salvo for a planned 10K genomes for the birds, dubbed the Bird10K project or B10K.

**Table 1:** **Vertebrate species with whole genome sequence published or posted**

	Lineage age (MYA)	No. species	No spp. with whole genome sequence
FISH	600	31564	60
AMPHIBIANS	300	6570	12
REPTILES	320	9002	19
BIRDS	150	10500	61
Mammals	220	5416	107
**SUM**		**63052**	**259**

Source: http://genome10k.org. (See also Koepfli et al., Ann Rev An Biosci, review in press for February 2015).

The reports released today from the open-access collaborative analyses of bird genome assemblies are an amazing harbinger for genome big-data collaborative projects, and the analyses and data here in many ways offer a refreshing preview of the hopes and perils of coming adventures for this, the Avian Genome Project, the G10K Project, and others like it.

At its onset, the Avian Phylogenomics Group looked at the biodiversity of birds and saw important questions and key advantages for moving forward with genome sequencing of numerous species. First of all, birds have off-loaded much of the ancestral transposon-based repeat families, a bane of mammal genome assemblies (~50% of the human genome is repetitive; while birds have but 5–10% repeats). Further, repeat family reduction, accompanied by massive segmental loss that has included more than 1000 genes, shrank the bird ancestral genome. Bird genomes are on the order of 1 Gbp versus 3 Gbp for most mammals and 6–9 Gbp for many amphibians, making birds eminently suited for such a massive sequencing project.

With 48 genomes now in hand, the group undertook extensive analyses from multiple angles and produced an overabundance of findings. The first of which was an incredibly robust phylogenetics tree for birds that resolved major ordinal splits, including many of those that occurred almost contemporaneously *circa* 66 MYA (1). Quite a feat since the avian hierarchy has been fraught with controversies and unresolved polytomies (i.e., ambiguous divergence nodes dividing species) in all but the most course-grained super-ordinal splits [9]. In several cases where branching order remains unresolved, the studies presented in this collection of papers provide evidence for incomplete lineage sorting, in which segregating polymorphism is differentially inherited among descendant lineages, a common occurrence among rapidly diverging species events. In the process the Avian Phylogenomics Group fine-tuned genome assembly, alignment and phylogenetic analysis algorithms, avoiding many artifacts that big-data comparative genomics studies face today and taking the field to a new level.

Moving from phylogeny to biology, their work uncovered many new and provocative candidate gene associations (gene expansions, contractions, selection signatures or modification) for avian characteristics, such as for vocal learning (cadherin 4-CDH4), for skeletal development in parallel with fight accommodation (Alpha-2-HS-glycoprotein [AHSG], associated with bone mineral density), for efficient high oxygen metabolism to power flight (gene loss of latent TGF-β binding protein 3-LTBP3, a critical lung gene in mammals), and for feather development (expansion of β-keratins to 1623 complete and 1084 incomplete gene copies and contraction of α-keratins relative to reptiles and mammals) [2]. Loss of hens’ —and other birds’— teeth seems to involve knock-out deletions of six enamel and dentin synthesis pathway enzyme coding genes. Faster divergence rates were seen in 15 genes involving plumage pigmentation, while re-organization of the opsin gene family offers a new blueprint for the amazing wavelength breadth and visual acuity in many birds, such as the incredible precision sight of *Pelicanus crispus*, the Dalmatian pelican, sequenced here, that makes a living diving for ocean fish.

In all, 35 reports have so far emerged from this avian genome sequence collection, and provide rich new genomic details about avian reproduction, sex determination, sexual adaptations, behaviors, endogenous retroviral footprints, genome contraction relative to reptiles and mammals, genome exchange breakpoints and ecological accommodations. This compendium represents the most extensive comparative genomics analysis produced for any vertebrate group so far.

In the face of the big bird advances, there remain formidable challenges to the Genome 10K reverie. NextGen sequencing technology, with a price tag approaching $1000 USD, provides a real bargain for reasonable coverage of a genome the size of the human genome (~3.0 Gbp) [10], but that $1000 sequencing product is not so good unless it *is* a human genome. The human genome has a reference sequenced to very high accuracy and contiguity to use as a framework, whereas most other species do not. Thus, to achieve the level of sequence completion desired, there is a need for more cost-effective technology to properly assemble short sequence contigs to long-range chromosomal contiguity without a reference. Budget constraints for the bird project were such that exceptional measures to achieve long-range contiguity could be applied only to a select subset of species. The community stands in urgent expectation of a solution to this bioinformatics dilemma for robust genome assemblies, for gene and genome feature annotations, for genome alignments, and for comparative analyses.

Further, the logistics of DNA transfer necessary for large-scale sequencing is severely impeded by permitting constraints for moving biospecimens internationally. Ironically, given the high value of information obtained by sequencing, regulatory processes constraining the movement of genomic DNA samples of threatened species— put in place to protect those species— may actually end up slowing conservation efforts. The Genome 10K Community of Scientists (G10KCOS) supports a rethinking and streamlining of these regulations now.

So where are we and will we ever hit 10,000 vertebrate genomes, let alone 10,000 bird genomes? Table 1 shows a near ten-fold increase in the number of species sequenced since G10K began 5 years ago, and the birds as a group make up just under 25%. If we continue at this rate of growth (10×/5 yrs.) we’d expect 2500–3000 species in 2019 and to hit the 10,000 mark a few years hence. Over 100,000 human genomes have already been sequenced, so with resolution of the issues discussed above, this seems achievable, and given the findings presented from just these few bird species, well worthwhile. Also encouraging is the emergence of several new sequencing consortia aimed at sequencing species of insects with human impact (Insect 5K), marine invertebrates (GIGA), snakes, fungi, microbes and plants.

## Conclusions

The Avian Phylogenomics Group’s achievements represent a beacon of hope that we shall move forward deliberately in the quest for vertebrate genome sequence assessment, analysis and release. The finished assemblies, annotations, gene orthologs, optical maps and more of the new bird genomes are archived and widely available in open access repositories (EBI, NCBI, DDBJ) for inspection and further analyses, and were released pre-publication in the *GigaScience* database, *Giga*DB (data described in [11]) allowing the entire community to begin their own investigations as soon as possible. We look forward to the day when nearly all vertebrate species will be curated in an online library of genomes. There, biologists of a coming generation will employ new tools for exploring the gene scripts that made the wonders of biological development and survival happen.

### Note from the Editors

*GigaScience*, *Genome Biology* and a number of BMC-Series journals are collecting a series of companion papers from the following series page: http://www.biomedcentral.com/series/avian.

## Endnotes

^a^SM1 Rationale for selection of species, sex, tissue sources, and bird collection details in Reference [1] Supplemental for a detailed narrative background description.

## Abbreviations

B10K: Bird 10,000 genomes project
G10K: Genome 10K project
G10KCOS: Genome 10K Community of Scientists
MYA: Million years ago.
